# Monitoring Corrosion of Steel Bars in Reinforced Concrete Structures

**DOI:** 10.1155/2014/957904

**Published:** 2014-01-16

**Authors:** Sanjeev Kumar Verma, Sudhir Singh Bhadauria, Saleem Akhtar

**Affiliations:** ^1^Civil Engineering Department, University Institute of Technology, Rajiv Gandhi Technical University, Airport Road, Bhopal, Madhya Pradesh 462036, India; ^2^Shri G.S. Institute of Technology and Science, Indore, Madhya Pradesh 452003, India

## Abstract

Corrosion of steel bars embedded in reinforced concrete (RC) structures reduces the service life and durability of structures causing early failure of structure, which costs significantly for inspection and maintenance of deteriorating structures. Hence, monitoring of reinforcement corrosion is of significant importance for preventing premature failure of structures. This paper attempts to present the importance of monitoring reinforcement corrosion and describes the different methods for evaluating the corrosion state of RC structures, especially hal-cell potential (HCP) method. This paper also presents few techniques to protect concrete from corrosion.

## 1. Introduction

Deterioration of concrete structures due to harsh environmental conditions leads to performance degradation of RC structures, and premature deterioration of structures before completing expected service life is major concern for engineers and researchers. Deterioration rate of structures depends on the exposure conditions and extent of maintenance. Corrosion, a result of chemical or electrochemical actions, is the most common mechanism responsible for deterioration of RC structures which is mainly governed by chloride ingress and carbonation depth of RC structures. Usually, there are two major factors which cause corrosion of rebars in concrete structures, carbonation and ingress of chloride ions. When chloride ions penetrate in concrete more than the threshold value or when carbonation depth exceeds concrete cover, then it initiates the corrosion of RC structures. If the corrosion is initiated in concrete structures, it progresses and reduces service life of the structures and rate of corrosion affects the remaining service life of RC structures. However, these severe environments can cause corrosion of reinforcement only if required amounts of oxygen and moisture are available at the rebar level in concrete structures [1].

**Figure 1:**
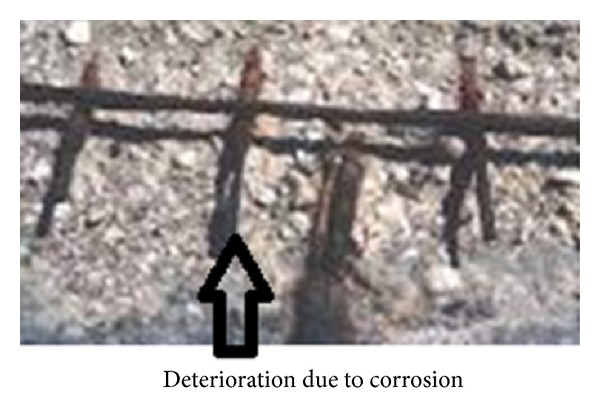
Deterioration resulting from corrosion.

**Figure 2:**
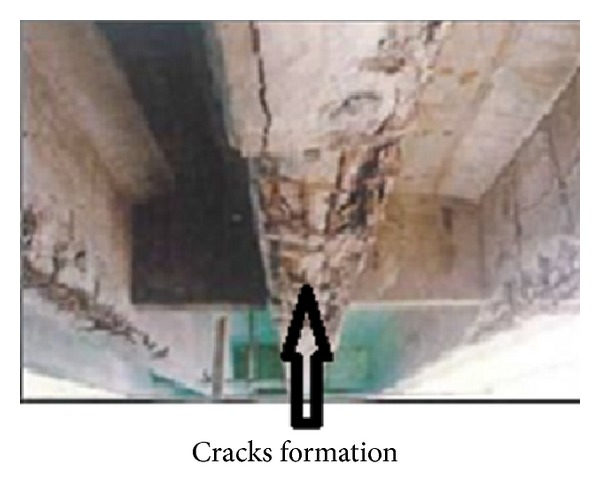
Cracks formation.

Corrosion of steel bars is the major cause of failure of concrete structures and about two tons of concrete is used per capita of the world population every year. Therefore, it has been realized that durable structures will reduce the cement consumption. Corrosion can severely reduce the strength and life of structures and in humid conditions pollutants from atmosphere percolate through the concrete cover and cause corrosion of steel. After the initiation of corrosion in reinforcing steel, products of corrosion expand and occupy a volume of about 6–10 times greater than that of steel resulting in the formation of cracks and finally in the failure of structures as shown in Figures 1 and 2.

Penetration of corrosion inducing agents such as chloride ions and carbon dioxide increased at the places of cracks, which further increases the corrosion [2]. Corrosion in concrete structures can be prevented by using low permeable concrete which minimizes the penetration of corrosion inducing agent, and the high resistivity of concrete restricts the corrosion rate by reducing the flow of current from anode to cathode [3].

## 2. Half-Cell Potential Method

Detection and evaluation of probability of corrosion in RC structures are essential. Proper corrosion monitoring of the concrete structures has been required for planning maintenance and replacement of the concrete structures. The most appropriate repair strategy can be selected for a distressed concrete structure by determining the corrosion status of reinforcing bars [4]. Repair of concrete structures without understanding the root cause of failure may be unsuccessful. If a cracked concrete patched without any treatment to the corroded steel, corrosion will likely continue and result in failure of patch work. Several methods for detecting corrosion activity discussed by authors in their previous paper [5] have been presented in Table 1.

**Figure 3:**
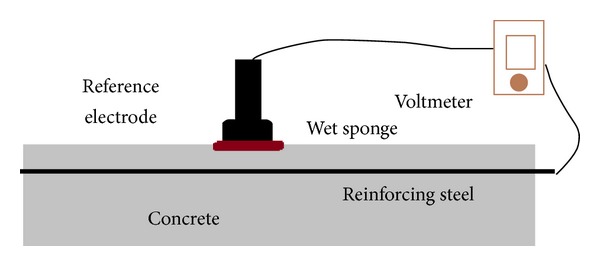
Setup of half-cell potential measurement.

There are several methods available for detecting and evaluating the corrosion in reinforcement steel as presented in Table 1. However, half-cell potential has been recognized by many researchers as the main method to detect the corrosion activity in RC structures [6]. In this method potential difference is measured between steel reinforcement and an external electrode with a voltmeter. The half-cell consists of a metal rod immersed in a solution of its own (Cu/CuSO_4_ or Ag/AgCl). The metal rod is connected with reinforcement steel by a voltmeter as shown in Figure 3. Some surface preparations including wetting to ensure good electrical connection are necessary. The main application of this method is in situ. External electrode and steel reinforcement are connected through a wet concrete cover as shown in Figure 3.

Interpretation of results of half-cell potential measurement for reinforced concrete structures required high skills and experience, as this only provides information regarding the probability of corrosion instead of rate and nature of corrosion [7]. Availability of oxygen, cover thickness, and concrete resistivity are few factors influencing the results of half-cell potential test. This method evaluates the potential difference on the exposed surface of concrete structures. The potential can be measured at any point on the surface or average of several measurements taken from different points on the same surface may be considered for evaluating the probability of corrosion. More negative value of measured half-cell potential indicates more probability of corrosion, as indicated in Table 2 according to ASTM C876 for Cu/CuSO_4_ half-cell.

**Figure 4:**
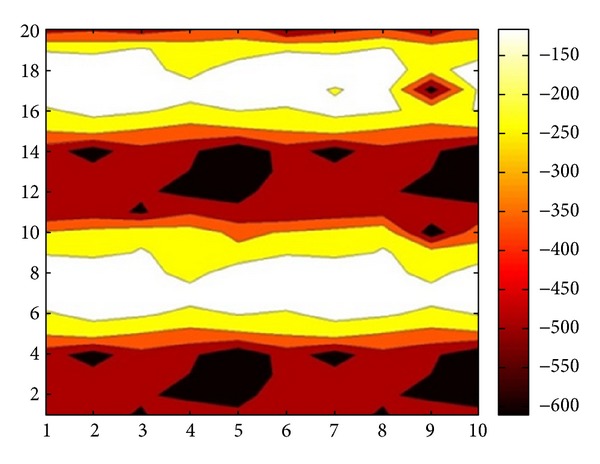
Half-cell potential contour.

This half-cell potential is also known as open circuit potential and is measured at several distinct points over a given area to be surveyed. Measured half-cell potential values can be used to plot a potential contour for the surface of reinforced concrete structure and this potential contour map as shown in Figure 4 can be used to evaluate the probability of corrosion at different points on the surface of the concrete structures. Portions of the structures likelihood of high corrosion activity can be obtained and identified by their high negative potentials.

## 3. Few Recently Conducted Corrosion Monitoring Activities

Several techniques have been reported in previous literatures that can be used for monitoring and evaluating the corrosion of rebars in concrete structures for diagnosing the cause and effect of corrosion. Few such studies performed by different researchers have been presented in Table 3.

## 4. Methods to Protect Structures from Corrosion

To increase the service life of RC structures, it is required to protect reinforcing steel completely from being corroded. Several chemical and mechanical methods are developed to prevent concrete structures from corrosion by retarding the corrosion rate and by controlling corrosion through reducing permeability of concrete and reducing the ingress of harmful ions such as oxygen and moisture, and some protective systems have been used in the form of coating. Different corrosion inhibitors and protecting systems have been discussed in Table 4.

## 5. Relative Limitations of Half-Cell Potential Method

Manually measuring potential values at different points on a large structure is tedious work. Therefore, automatic evaluating method is required. Half-cell potential measurements are widely used in structural engineering to assess the likelihood of corrosion. HCP measurements are found to be associated with several practical limitations such as (1) establishing connection with reinforcement, especially in structures with large concrete cover, (2) properly wetting the concrete cover for establishing proper connection between reference electrode and reinforcement, and (3) availability of oxygen, cover thickness, and concrete resistivity which can influence the results of half-cell potential test.

HCP method only provides the evaluation of the point likely to be corroded and no assessment of the corrosion rate. Half-cell potential values are indicative of the probability of corrosion activity of reinforcement located beneath the reference electrode only if the steel rebars are electrically well connected to the voltmeter. Half-cell potential method cannot provide reliable results with epoxy coated reinforcement or with coated concrete surfaces. Moist or wetting condition of concrete can influence the results of half-cell potential method, or it is important to assure the sufficient wetting of concrete to complete the setup for valid half-cell potential measurement. If measured value of the HCP varies with time, prewetting of the concrete is required. It is essential to thoroughly wet the concrete surface and allow sufficient time for the moisture to penetrate the surface layer to stabilize the potential. ASTM C-876 emphasizes that if the measured value of half-cell potential changes with time surface of concrete should be wet for at least 5 min.

It has been observed from literature that results of HCP mapping required careful interpretation. To interpret HCP data, factors such as variation in moisture content, chloride content, and concrete electrical resistance are required to be considered as all these parameters have a significant influence on the readings.

The major drawback is that HCP requires a localized breakout of the concrete cover to provide an electrical connection to the steel reinforcement. HCP results are highly influenced by the composition of the deteriorated concrete. Therefore, interpretation criteria might be different for different deterioration types. Shortcomings of HCP measurements result from the fact that the potentials are measured not near rebars but on concrete surface. Compensation is required to get more reliable results.

## 6. Conclusion 

Failure of concrete structures due to corrosion of embedded rebars is a major problem causing significant loss of money and time. Hence, there is a need to fully understand the root causes of failure before the repairing for effective remediation. An effective method to measure corrosion is a fundamental requirement for planning maintenance, repairing, and removal for reinforced concrete structures. Information regarding corrosion state required three parameters: half-cell potential, concrete resistivity, and corrosion current density. Corrosion rate in a concrete structure is governed by several parameters such as moisture content, availability of oxygen, and temperature. So, for better results it is necessary to repeat corrosion rate measurement in regular time interval.

Half-cell potential measurement is the most widely used technique for the evaluation of corrosion of steel in concrete. However, in interpreting the data environmental factors should be taken into account. For interpretation of half-cell potential readings, it requires precise understanding of corrosion protection mechanisms and good knowledge and experience in half-cell potential mapping. In present research it has been observed that half-cell potential measurements are useful in the following purposes:

1. to assess the corrosion condition of the reinforcement by locating corroded bars,
2. for the condition assessment of a concrete structure,
3. to locate and decide the position of further detailed destructive and nondestructive testing,
4. evaluate the efficiency of repair work through corrosion state monitoring of repaired concreter structures.

In concrete with low resistivity potential distribution on surface represents potential at steel concrete interface. For better results interpretation of potential readings can be done in accordance with resistivity. With increase in concrete cover difference between surface and interface potential increases.

Content of this paper can be utilized to understand the principal of half-cell potential method, to plan investigation of corroded structures, and to select suitable corrosion monitoring technique.

## Figures and Tables

**Table 1:** Methods for evaluating corrosion in concrete structures [5].

S. no.	Method	Advantages	Limitations	Principle
1	Galvanostatic pulse method	Measures half-cell potential and electrical resistance simultaneously	Unstabilized readings	Based on the polarization of rebar by means of small constant current
2	Linear polarization resistance (LPR)	Rapid and requires only localized damage, more detailed information	Measurements are affected by temperature and humidity	Electrical conductivity of fluid can be related to its corrosiveness
3	Half-cell potential	Simple, portable, results in the form of equipotential contours	Needs preparation, saturation required, not very accurate, and time consuming	Electric potential of rebars is measured relative to half-cell and indicates probability of corrosion
4	Time domain reflectometry (TDR)	More robust, easy, and locates corrosion and identifies extent of damage	Less sensitive	By applying a sensor wire alongside of the reinforcement a transmission line is created. Physical defects of the reinforcement will change the electromagnetic properties of the line
5	Ultrasonic guided waves	Identify location and magnitude of corrosion	Not very reliable	Based on propagation of ultrasonic waves
6	X-ray diffraction and atomic absorption	Simple and reliable	Hazardous	Intensity of X-ray beams reduces while passing through a material

**Table 2:** Presents criteria according to ASTM C876 for Cu/CuSO_4_.

S. no.	Half-cell potential (mV)	Probability of corrosion
1	>−200	10%
2	−200 to −350	50%
3	<−350	90%

**Table 3:** Several recent corrosion studies.

Reference	Study performed	Significant observations	Comments
Pour-Ghaz et al., 2009 [7]	Presented a tool for the interpretation of the results of half-cell potential measurement. It relates half-cell potential values to the probability of corrosion through concrete resistivity, cover thickness, temperature and anode to cathode ratio. A model is developed by solving Laplace's equation, relating corrosion current with average potential on the surface, potential difference on the concrete surface, temperature, resistivity, and concrete cover.	In concrete with low resistivity potential distribution on surface represents potential at steel concrete interface. For better results interpretation of potential readings can be done in accordance with resistivity. With the increase in concrete cover difference between surface and interface potential increases.	More realistic results can be obtained by considering availability of oxygen and increasing the test points. More experimental validation of the model is required to increase the confidence.

Song and Saraswathy, 2007 [6]	Reviewed several electrochemical and nondestructive testing methods for the assessment of corrosion in concrete structures.	Combining several techniques can provide more information about corrosion state of steel bars. An integrated monitoring system for new and existing concrete structures can reduce inspection cost.	Presented methods are useful to monitor corrosion in concrete structures and all these reviewed methods can be used to develop more accurate and better techniques for monitoring corrosion.

Ahmad, 2003 [3]	Reviewed mechanism of corrosion, corrosion monitoring techniques, and methodologies to predict the remaining service life of structures. Observed that corrosion rate is affected by pH of electrolyte, availability of oxygen, capillary water, and concentration of FE^2+^ in the concrete near the reinforcement.	Information regarding corrosion state required three parameters half-cell potential, concrete resistivity, and corrosion current density.	Presented all the aspects of corrosion, and may be useful for understanding the corrosion theory, progress of corrosion, factors affecting corrosion, monitoring techniques and for predicting service live of structures.

Bjegovic et al., 2000 [2]	Described different corrosion monitoring techniques such as half-cell potential measurement, macrocell current measurement, linear polarization method, Geocor 6, electrochemical impedance spectroscopy, Galvanostatic pulse method, and scanning reference electrode method.	Nondestructive methods for measuring corrosion are advantageous as measurements can be done over entire structure, provide fast results, and are inexpensive.	Presented overview of several nondestructive methods with their relative advantages and disadvantages based on experiences and interpretation of results. It is a useful study covering almost all the present corrosion measuring techniques.

Carino, 1999 [4]	Presented an overview of corrosion process and nondestructive evaluation techniques such as half-cell potential method, concrete resistivity test, and the linear polarization method.	Corrosion rate in a concrete structure is governed by several parameters such as moisture content, availability of oxygen, and temperature. So, for better results it is necessary to repeat corrosion rate measurement in regular time interval.	A useful review has been presented by considering the behavior of electrolytic cells.

So and Millard, 2007 [8]	Presented Galvanostatic pulse transient technique for evaluating the corrosion rate in reinforced concrete structures and also presented the advantages of this technique over linear polarization (LPR) method.	Corrosion rates calculated from Galvanostatic pulse transient technique are generally higher than those evaluated from LPR technique.	It is a useful study presenting a relatively more reliable technique for measuring corrosion rate in RC structures.

Pradhan and Bhattacharjee, 2009 [9]	Discussed results of a study conducted on concrete specimens with different cement, steel, and varying water/cement ratios. Specimens are subjected to 3% sodium chloride solution and half-cell potential measurements were carried out to evaluate corrosion activity.	Critical chloride content causing corrosion initiation is influenced by steel type, cement type, and w/c ratio. Found half-cell potential as a parameter indicating rebar corrosion initiation in chloride contaminated concrete.	It has been observed from this study that corrosion initiation time is influenced by the rate of ingress of chloride ions and depassivation of protective passive film.

Hussain and Ishida, 2012 [1]	Performed multivariable laboratory experiments to evaluate effect of oxygen on reinforcement corrosion under different environmental conditions and also explained half-cell potential measurement in different conditions such as submerged exposure condition and under cyclic wetting—drying exposure.	It was observed that oxygen is an influencing factor for corrosion only for concretes placed completely under water.	Results of this analysis can be used for calibrating half-cell potential measurements performed under water.

Cairns and Melville, 2003 [10]	Performed nondestructive electrochemical measurements of corrosion to evaluate effect of protective coatings on the reliability of these tests.	It has been observed from results that half-cell potential measurements were not affected significantly by coating.	Useful study to evaluate reliability of corrosion monitoring techniques.

Elsener, 2001 [11]	Discussed about application and limitations of half-cell potential mapping for assessing reinforced concrete structures to evaluate repair work. Repairs include replacement of chloride contaminated concrete, electrochemical chloride removal, electrochemical realkalization and application of corrosion inhibitors.	For interpretation of half-cell potential readings, it requires precise understanding of corrosion protection mechanisms and good knowledge and experience in half cell potential mapping.	A useful study explaining half-cell potential mapping and effect of corrosion repairing over the results provided by half-cell potential method.

Parthiban et al., 2006 [12]	Carried out simultaneous potential measurements on different points on concrete slab, using computer based I/O cards and also developed software based on ASTM C-876 for interpretation of measured values.	Among the various electrochemical methods potential measurement has been the mostly used field technique for detecting corrosion activity in steel. Manually measuring half-cell potential values is a tedious job on a large structure, so an automatic system to evaluate the half-cell potential values is present.	An automated useful method to evaluate half-cell potential at different points on a large structure simultaneously is present. This method can reduce time required to evaluate potential values at different points for monitoring the corrosion.

Moon and Shin, 2006 [13]	Studied corrosion evaluation of the steel bars embedded in underwater concrete. Performed accelerated corrosion tests on three series of reinforced underwater concrete with different admixtures in different conditions.	It has been observed that specimens casted in seawater develop early corrosion of steel bars. Among all the specimens, in OPC manufactured concrete corrosion rate is fastest and exceeds threshold value earlier than other specimens. Mineral admixtures are more effective in delaying the development of corrosion in underwater concrete.	A careful study on antiwashout underwater concrete to evaluate effect of different admixtures on corrosion of steel bars.

Poursaee and Hansson, 2009 [14]	Described pitfalls in assessment of chloride induced corrosion through electrochemical methods. Factors influencing the results of electrochemical processes are found to make more measurements in short period to reduce the costs, choosing appropriate electrochemical method, and laboratory tests are usually conducted on young and immature concrete.	Results of electrochemical assessment may not represent actual condition of rebars.	Explained the pitfalls in electrochemical assessment of chloride induced corrosion of steel, which can be utilized to regulate the results of measurements.

Soleymani and Ismail, 2004 [15]	Performed a study to estimate the corrosion activity of steel bars embedded in two types of concrete specimens, ordinary and high performance, applying different corrosion measurement methods. Methods applied are half-cell potential, linear polarization method, Tafel plot, and other chloride content methods.	Results indicated that all these method would assess the same level of corrosion in only 24% of specimens.	Presented a useful comparison between different corrosion measurement methods. This study can be used by researchers to select better corrosion monitoring technique.

Ahn and Reddy, 2001 [16]	Performed accelerated corrosion test to evaluate durability of marine concrete structures subjected to fatigue loading with different water cement ratios. Ultimate strength testing followed by half-cell potential measurement and crack investigations has been performed.	Deterioration is faster under fatigue loading than static loading. Durability decreased with increase in water cement ratio.	Presents significant findings about the effect of fatigue loading and water cement ratio over the durability and life of the structures.

Elsener, 2002 [17]	Studied effect of conductivity and cover depth on potential and macrocell current distribution. Also, discussed consequences of monitoring corrosion through half-cell potential mapping and polarization measurement technique on locally corroded rebars.	Low electrolyte conductivity and cover make it possible to locate anode of the macrocell by potential measurements.	Discussed about influence of macrocell corrosion on corrosion monitoring.

Alhozaimy et al., 2012 [18]	Performed laboratory experiments to evaluate half-cell potential, corrosion current, and concrete resistivity over chloride contaminated concrete specimens, to investigate the phenomenon of high corrosion at intersection of steel rebars in the wall footing.	Observed that experimental measurements are higher at intersection of steel bars in comparison with the areas between them. This high corrosion rate is found to be due to coupled effects of corrosive binding wire materials, electrical connectivity, reduction in centre to centre spacing of steel rebars, and poor concrete microstructures.	Phenomenon reported in this paper is new and interesting. More and extensive research is required to understand the effect of all factors influencing the corrosion at intersection of steel rebars.

Duong et al., 2013 [19]	Performed half-cell potential and corrosion current density test on concrete specimens to monitor corrosion activity. This corrosion activity had been monitored to evaluate the effect of leaching on carbonation and corrosion initiation of steel bars.	Observed that with the increase in leaching exposure carbonation depth also increases. Replacing cement partially with fly ash reduces the resistance against carbonation and leaching.	Presents the performance of half-cell potential measurement and corrosion current density to detect corrosion due to leaching activity. It has been observed that suitable test methods are required.

Sadowski, 2010 [20]	Describes linear polarization and four point Wenner resistivity methods to evaluate corrosion rate without making a direct connection to the reinforcement.	Observed that short circuit influence of embedded steel can be used to evaluate the rate of corrosion on the surface of the bars.	More validation of methods is required on concrete with wider range of resistivity.

Jung et al., 2003 [21]	Half-cell potential and linear polarization measurements have been performed for one year to evaluate the parameters affecting the corrosion rate. Measurements have been made to predict the remaining service life of land concrete affected from steel corrosion.	Quantitative polarization method provides more precise results than those of half-cell potential method in evaluating the corrosion activity.	Comparison between methods helps researchers to select better techniques for evaluating residual service life of structures.

Lai et al., 2013 [22]	Presented a new technique to investigate corrosion of steel bars in concrete using ground penetrating radar (GPR) and modified half-cell potential method. Attempted to measure potential difference with two moving probes and making no connection with steel bars.	Results show that both GPR and modified HCP methods can measure electrochemical corrosion process.	More researches are required to relate laboratory results with real time structures

Leelalerkiet et al., 2004 [23]	Performed half-cell potential measurements to estimate corrosion of reinforcing steel bars embedded in concrete slabs under cyclic wet and dry exposures. Influence of void over potential distribution and current distribution has also been investigated.	Observed from results that half-cell potential is marginal successful In the void specimens half-cell potential values required compensation for more reliable results.	Useful study to demonstrate corrosion estimation in both intact and void specimens.

Faber and Sorensen, 2002 [24]	Discussed the application of half-cell potential measurements to evaluate the probability of corrosion and repair after 50 years. This is explained on a corroded concrete structure.	It has been observed that half-cell potential measurements may be utilized to update the probability of corrosion.	Provided a study on the utilization of half-cell potential method.

Hussain, 2011 [25]	Investigated underwater half-cell corrosion potential in submerged RC structures and compares with various other relative humidity conditions.	Half-cell potential values for submerged underwater RC structures are not representing actual corrosion rate and these values are required to be calibrated using the experiment results of this research.	This study enables researches to perform underwater corrosion measurement for evaluating condition of submerged RC structures.

**Table 4:** Techniques to protect concrete from corrosion.

Protective techniques	Reference
Fly ash increased the corrosion resistance of concrete by reducing porosity of concrete porosity, which decreases penetration rate of harmful ions.	Xu et al., 2012 [26]
Super-plasticizers and mineral admixtures like fly ash, granulated blast furnace slag, and pozzolanic materials reduce the corrosion rate.	Maslehuddin et al., 1992 [27]
Use of low-nickel stainless steel rebars reduces corrosion rate by providing high alkaline concrete pore solution	Criado et al., 2011 [28]
Penetrating amino alcohol corrosion inhibitor reduces the steel corrosion.	Jamil et al., 2005 [29]
Calcium nitrite based corrosion inhibitor reduces the carbonation depth	Sideris and Savva, 2005 [30]
Calcium nitrite based inhibitor improves the chloride threshold value.	Ann et al., 2006 [31]
Benzotriazole as a corrosion inhibitor improves corrosion resistance.	Ann et al., 2006 [31]
Polyvinylpyrrolidone improves corrosion resistance of concrete when added.	Gürten et al., 2005 [32]
Alkylamino alcohol increases the corrosion resistance.	Morris and Vázquez 2002 [33]
Fusion bonded epoxy coated (FBEC) steel bars are beneficial in decreasing corrosion.	Al-Dulaijan et al., 2012 [34] Darwin and Scantlebury, 2002 [35]
Alkanolamine based corrosion inhibitor with inorganic coating.	Batis et al., 2003 [36]
Steel bars coated with DINITROL AV 30 shows good corrosion resistance.	Monticelli et al., 2000 [37]
Use of double combination of calcium nitrite and ground granulated blast furnace slag (GGBFS), and triple combination of calcium nitrite, silica fume, and fly ash/GGBFS protect concrete exposed to severe corrosive environments.	Civjan et al., 2005 [38]
Aminoalcohol based mixed (organic/inorganic) inhibitors, when used as admixture or as a repair product, reduces the rate of corrosion.	Wombacher et al., 2004 [39]
ZnO reduces the concrete porosity and chloride content at rebar level and reduces the corrosion	de Rincón et al., 2002 [40]
By providing high chromium steel, corrosion rate can be decreased.	Nachiappan and Cho, 2005 [41]
CFRP laminates reduce the expansion caused by corrosion and also control the corrosion rate by decreasing the loss of mass.	Badawai and Soudki, 2005 [42]
